# Clinical analysis of 12 cases of acute exogenous lipoid pneumonia in children

**DOI:** 10.3389/fped.2026.1809747

**Published:** 2026-04-28

**Authors:** Shuai Liu, Zexi Wang, Lijing Cao, Meixian Xu

**Affiliations:** 1 Hebei Children’s Health and Disease Clinical Medical Research Center, Department of Intensive Care Medicine, Hebei Medical Key Discipline, Hebei Children’s Hospital, Hebei, Shijiazhuang, China; 2 Hebei Children’s Health and Disease Clinical Medical Research Center, Department of Neurorehabilitation, Hebei Children’s Hospital, Hebei, Shijiazhuang, China

**Keywords:** accidental, bronchoscopy, corticosteroid, exogenous lipid pneumonia, oil and fat substances

## Abstract

**Objective:**

To explore the clinical characteristics and diagnosis and treatment process of acute exogenous lipoid pneumonia (AELP) in children, in order to improve treatment efficacy and prognosis.

**Method:**

This study retrospectively analyzed the clinical data and treatment process of patients with AELP admitted to the Intensive Care Medicine Department of Hebei Children’s Hospital from January 2017 to October 2025.

**Results:**

1.This study collected 12 patients, according to the interval between exposure to oil substances and hospitalization,patients with a delay of more than 12 h were classified as the delayed visit group, while those with a delay of less than or equal to 12 h were classified as the early visit group.Compared with the early visit group, the delayed visit group had significantly lower oxygenation score, higher inflammatory markers (WBC, CRP, LDH), and longer hospital stays (all *P* < 0.05). 2.The oxygenation score of children with hospitalization time >7 days was lower than that of children with hospitalization time ≤ 7 days(*P* < 0.05). Eleven of 12 patients recovered; follow-up imaging showed complete resolution within 2 weeks to 3 months.

**Conclusion:**

Acute exogenous lipoid pneumonia (AELP) in children is caused by accidental inhalation of lipids.In this case series, a longer interval between oil exposure and hospitalization (>12 h) was associated with a higher likelihood of secondary infection, greater treatment difficulty, and longer hospital stay.Monitoring oxygenation scores upon admission might serve as a preliminary predictor for longer hospital stays, pending validation in larger studies. Fiberoptic bronchoscopy combined with corticosteroid and nebulization therapy was used, and most children had a good prognosis.

## 1 Introduction

Acute exogenous lipoid pneumonia (AELP) is a localized or diffuse pulmonary inflammatory response and pathological change caused by inhalation exposure to oily substances (1). Its clinical manifestations are similar to other diseases, usually manifested as difficulty breathing or coughing. The condition changes rapidly, and in severe cases, pulmonary edema, acute respiratory distress syndrome, etc. can occur. In adults, it can be caused by improper use of lipid-containing drugs, occupational exposure, etc. Children are often caused by accidental aspiration of lipids (2–4). According to several studies (5, 6), there are more reports related to adults, but fewer for children. The diagnosis of AELP still poses challenges in clinical practice. Its non-specific respiratory symptoms often lead to misdiagnosis as common pneumonia, although pathological biopsy is a definitive diagnostic method, it is difficult to perform in pediatrics. Therefore, diagnosis is usually based on a clear contact history and characteristic imaging. However, obtaining a clear contact history is not always easy, especially in infants and young children. In addition, there is currently no unified diagnostic standard for pediatric AELP. Therefore, it is crucial to summarize the clinical characteristics, diagnostic clues, and treatment experience of pediatric cases.To further enhance the vigilance and diagnosis and treatment level of pediatric AELP among clinical physicians, this study retrospectively analyzed the clinical data of 12 cases of pediatric AELP, summarized its etiology, clinical manifestations, imaging characteristics, diagnosis and treatment process, and prognosis, aiming to strengthen the understanding of the disease and provide reference for early diagnosis and reasonable intervention.

## 2 Subjects and methods

### 2.1 Research subjects

Retrospective analysis of 12 cases with acute exogenous lipoid pneumonia admitted to the Intensive Care Medicine Department of Hebei Children's Hospital (Hebei, China) from January 2017 to October 2025,including 7 males and 5 females, all of whom had relatively complete clinical medical records. Although pathological biopsy is considered a definitive diagnostic method, it is rarely performed in children due to its invasiveness.Therefore, the diagnosis in clinical practice relies primarily on exposure history, imaging findings, and exclusion of other pulmonary diseases. The inclusion criteria for this study were: (1) A clear history of exposure to oil substances (ingestion or inhalation); (2) Clinical manifestations include fever, cough, shortness of breath, vomiting, etc; (3) Abnormal changes in chest imaging; (4) Excluding underlying diseases such as tuberculosis and bronchopulmonary dysplasia.; (5)Clear evidence of pulmonary inhalation, defined as the presence of at least one of the following conditions: acute respiratory symptoms occurring within a few hours after exposure; The chest CT findings are consistent with aspiration pneumonia; Oil secretion in bronchoalveolar lavage fluid under bronchoscopy.Exclusion criteria: (1) History of respiratory infections such as fever, cough, etc. prior to exposure to oily substances; (2) Previous underlying diseases such as bronchopulmonary dysplasia and primary ciliary dyskinesia; (3) Those with incomplete case information. To evaluate the impact of delayed visits on clinical outcomes, patients were divided into two groups based on the interval between exposure to oily substances and admission: the delayed visit group (interval > 12 h, *n* = 7) and the early visit group (interval ≤ 12 h, *n* = 5). The deadline was selected based on the median interval (12.9 h) of the study population to compare early and late stage performance.

### 2.2 Research methods

#### 2.2.1 Clinical data collection

Retrospective analysis of 12 cases of AELP, collecting and recording the sex, age, pre onset disease course, clinical manifestations (such as fever, cough, vomiting, changes in consciousness, etc.), physical examination at admission (such as nasal flaring, positive chest retractions, pulmonary wet rales, etc.), blood routine examination,CRP,blood biochemistry, blood gas analysis, chest imaging, length of hospital stay, treatment history, prognosis, and other related data.

#### 2.2.2 Auxiliary examination data

After admission, peripheral venous blood was collected from all patients for blood routine tests(including white blood cell count), CRP, liver and kidney function (including alanine aminotransferase [ALT], aspartate aminotransferase [AST], blood urea nitrogen [BUN], and creatinine), myocardial enzymes(including creatine kinase [CK],creatine kinase-MB [CK-MB] and lactate dehydrogenase[LDH]),and coagulation function.Radial artery blood was extracted for blood gas analysis.Sputum culture, blood culture(using both aerobic and anaerobic bottles), and bronchoalveolar lavage fluid culture were performed to identify potential bacterial pathogens (e.g., *Acinetobacter baumannii*, *Staphylococcus aureus*). Chest imaging examinations were performed.The oxygenation score, defined as the PaO₂/FiO₂ ratio (mmHg), was calculated from arterial blood gas analysis at admission.

### 2.3 Statistical methods

SPSS 22.0 statistical software was used to perform statistical processing on the data. Due to the small sample size (*n* = 12), the normality of continuous variables was assessed using the Shapiro–Wilk test. Because not all variables followed a normal distribution, data are presented as median with interquartile range (IQR). For comparisons between two independent groups, the Mann–Whitney U test (non-parametric) was applied. A two-tailed *P* < 0.05 was considered statistically significant.

## 3 Results

### 3.1 General information

This study included a total of 12 children with AELP, all patients met the criteria for pulmonary aspiration, including 7 males (58.3%) and 5 females (41.7%), with an onset age of 4-48 months and an average age of 23.3 ± 12.2 months. Among them, 9 cases (75.0%) mistakenly consumed sewing machine oil, 2 cases (16.7%) mistakenly consumed mosquito repellent oil, and 1 case (8.3%) mistakenly consumed kerosene. The youngest case was mistakenly fed by his older brother. The interval between contact and medical treatment is 2-30 h, with an average of 12.9 ± 7.1 h. The hospitalization time is 5-20 days, with an average of 10.3 ± 4.3 days, as shown in Table 1.

**Table 1:** Clinical data of 12 pediatric patients.

Case number	Age (months)	Sex	Contact time to medical treatment (hours)	Cough	Fever	Vomit	Shor-tness of breath	Change of conscio-usness	Chest pain	Pulm-onary rales	Sputum culture	Alveolar lavage fluid culture	Number of bronchoscopy examinations	Hospitali-zation time (days)	Outcome
Case 1	4	male	5	+						−	−	−	1	8	recovered
Case 2	14	male	2			+				−	−	−	1	5	recovered
Case 3	24	female	12		+		+		+	dry	−	−	1	9	recovered
Case 4	48	male	24	+	+		+	+		wet	−	+	2	10	recovered
Case 5	35	female	16	+	+					wet	+	−	2	7	recovered
Case 6	12	male	14	+		+	+			dry	−	+	2	10	recovered
Case 7	26	female	26	+	+		+			wet	−	+	4	20	recovered
Case 8	19	male	30	+	+	+	+	+		dry and wet	+	+	3	15	death
Case 9	37	female	10							−	−	−	1	14	recovered
Case 10	15	male	9							−	−	−	1	6	recovered
Case 11	26	male	13	+			+			−	−	−	1	12	recovered
Case 12	20	female	20	+	+	+	+			wet	−	+	1	8	recovered

Regarding potential confounding factors, the distribution of oil exposure types was similar between the two groups of children: in the delayed visit group, sewing machine oil accounted for 5 out of 7 cases (71.4%), and in the early visit group, it accounted for 4 out of 5 cases (80.0%). Five patients (41.7%) were given mechanical ventilation and all belonged to the delayed visit group. Among them, 4 patients had a hospital stay of more than 7 days, and 1 patient (the only death) had a hospital stay of 15 days.

### 3.2 Clinical manifestations and physical examination

The main symptoms of the 12 children were cough, fever, vomiting, and shortness of breath. Among them, there were 8 cases of cough (66.7%), 6 cases of fever (50.0%), the highest fever peak was 39.4 ℃, 4 cases of vomiting (33.3%), 7 cases of shortness of breath (58.3%), the highest respiratory rate was 78 breaths per minute, 2 cases of consciousness change (16.7%), and 1 case of chest pain (8.3%). Three cases (25.0%) had wheezing sounds and prolonged exhalation on lung auscultation, five cases (41.7%) had wet rales on lung auscultation, and five cases (33.3%) had no obvious positive signs in the lungs, as shown in Table 1.

### 3.3 Auxiliary examination results

When 12 children were admitted, their white blood cell count ranged from 10 × 10⁹/L to 30 × 10⁹/L,mainly with elevated neutrophils,CRP fluctuated between 16 and 134 mg/L, oxygenation fraction fluctuated between 149-289mmHg, and LDH fluctuated between 209-498U/L.There were 7 cases in the delayed visit group, and ≤12 h in the early visit group, with 5 cases. As shown in Table 2, all measured parameters differed significantly between the two groups *(P* < 0.05). In the delayed visit group, 2 cases had positive sputum cultures (1 case of *Acinetobacter baumannii* and 1 case of *Staphylococcus aureus*), and 5 cases had positive bronchoalveolar lavage fluid cultures (3 cases of *Acinetobacter baumannii* and 2 cases of *Staphylococcus aureus*). All of these children experienced fever, elevated inflammatory markers were monitored, and antibiotic treatment was given, clinically supporting infection rather than colonization.In the early visit group, all cultures were negative after admission, as shown in Table 1.

**Table 2:** Comparison of clinical data between two groups of pediatric patients.

Group	Countdown	White blood cell count ( × 10^9^/L)	CRP（mg/L）	LDH（U/L）	Oxygenation score(mmHg)	Hospitalization time (days)
Delayed visit group	7	18.0 (14.0–23.0)	68.0 (57.0–89.0)	308.0 (287.0–326.0)	180.0 (158.0–197.0)	12.0 (10.0–15.0)
Early visit group	5	12.0 (11.0–14.0)	20.0 (19.0–21.0)	221.0 (210.0–230.0)	245.0 (218.0–287.0)	8.0 (6.0–8.0)
Z		−2.149	−2.807	−2.809	−2.407	−2.117
*P*		0.032	0.005	0.005	0.016	0.034

**Figure 1:**
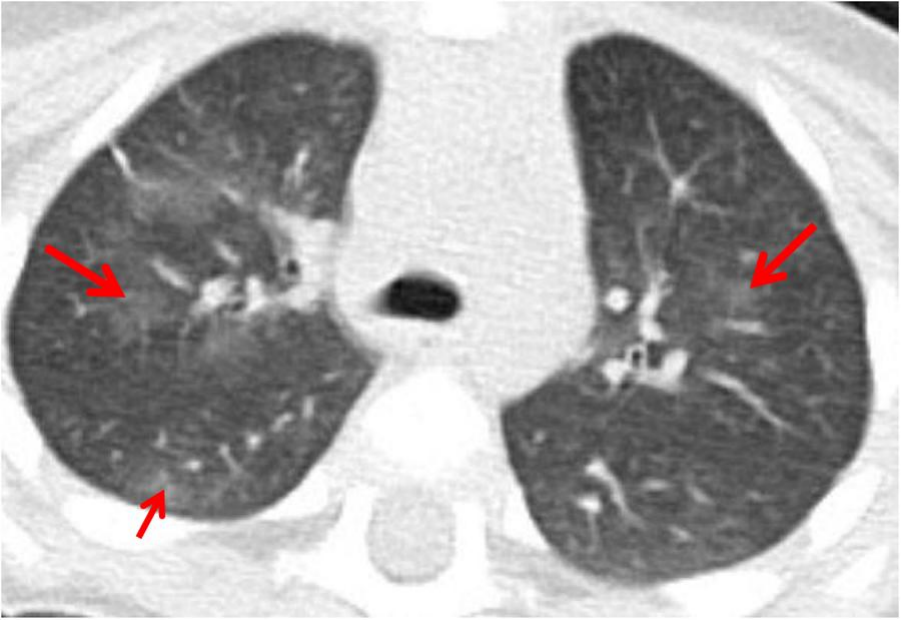
Chest CT findings in a 24-month-old female with acute exogenous lipoid pneumonia following accidental sewing machine oil ingestion.the patient presented to the hospital 12 h after exposure with fever and shortness of breath.Chest CT (lung window) shows patchy consolidation and ground glass opacities (arrows) in both lower lobes.

**Figure 2:**
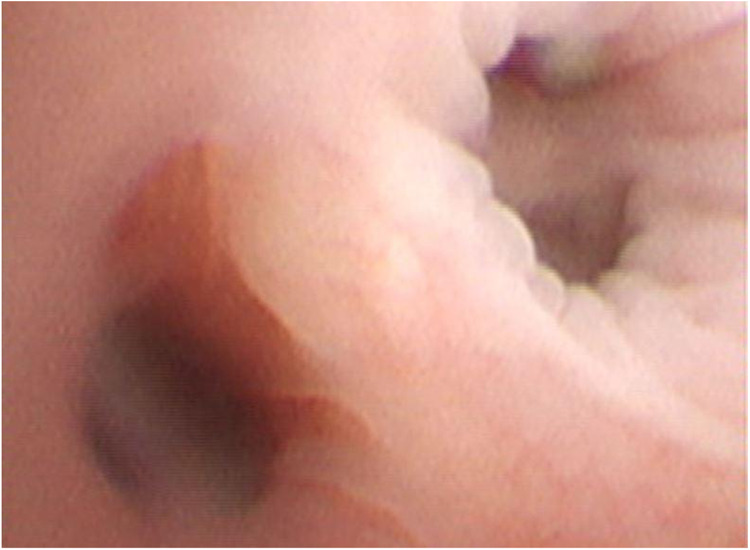
Bronchoscopic findings of the same patient.the image shows diffuse mucosal congestion and edema in the main bronchus. A large amount of gray white viscous secretions can be seen inside the lumen, with thickened mucosa and the appearance of red and white spots.

All patients underwent chest CT examination within 24 h of admission, and all results showed exudation or consolidation changes (See Figure 1). Six cases (50.0%) had lesions involving both lower lungs. After admission, all patients underwent fiberoptic bronchoscopy examination, which showed congestion and edema of the tracheal and bronchial mucosa. There were many gray white secretions in the lumen, and the mucosa was thick and white with red and white spots (See Figure 2). Among them, 7 cases (58.3%) only underwent one examination, and 5 cases (41.7%) underwent 2-4 examinations. All 5 cases were in the delayed visit group, as shown in Table 1.

There were 9 children with a hospital stay of more than 7 days, and their white blood cell count, CRP, and LDH were all higher than those of ≤7 days, but the differences were not statistically significant (*P* > 0.05). The oxygenation score is less than or equal to 7 days, and the difference is statistically significant (*P* < 0.05), as shown in Table 3.

**Table 3:** Comparison of auxiliary examinations for children with different hospitalization times.

Group	Countdown	White blood cell count ( × 10^9^/L)	CRP（mg/L）	LDH（U/L）	Oxygenation score (mmHg)
Hospital stay >7 days	9	15.0 (12.0–19.0)	67.0 (29.0–89.0)	292.0 (214.0–326.0)	189.0 (158.0–218.0)
Hospital stay ≤7 days	3	11.0 (9.0–14.0)	19.0 (16.0–20.0)	221.0 (209.0–230.0)	287.0 (245.0–289.0)
Z		−1.235	−1.907	−1.599	−2.352
*P*		0.217	0.057	0.110	0.019

### 3.4 Treatment and outcome

All patients were subjected to fiberoptic bronchoscopy bronchoalveolar lavage within 1-2 days after admission, and oily substances were observed in the lavage fluid. As described under the microscope, budesonide (Astra Zeneca Pty Ltd, production batch number: 3231452) was injected through bronchoscopy, and 5 patients were given 2-4 times of bronchoscopy lavage. After admission, all patients were given intravenous corticosteroid infusion. Five patients were treated with methylprednisolone (Pfizer Manufacturing Belgium NV, production batch number: W59745) at a dose of 2 mg/(kg day) for 3 days. Seven patients were treated with methylprednisolone at a dose of 2 mg/(kg·time) once every 12 h for 3 days, and then reduced to once a day for 3 days before discontinuation. Five cases of children with rapid breathing upon admission were given tracheal intubation and assisted breathing with a ventilator, as well as 2 g/kg of human immunoglobulin (produced by Hualan Bioengineering Co., Ltd., batch number: 201712047). All patients were treated with budesonide combined with salbutamol nebulization therapy. All patients were treated with antibiotics.

11 patients were cured and discharged, and 1 patient died clinically. The cure criteria are clinical rehabilitation and improvement in chest imaging examination.All patients were discharged in good general condition, with clear consciousness, good reactions, and no symptoms such as fever, cough, or shortness of breath. Physical examination of the lungs showed no significant abnormalities. Follow up was conducted 2 weeks to 3 months after discharge based on clinical needs and patient availability. Patients with more severe initial or residual symptoms received early follow-up (2-4weeks), while patients with symptom relief upon discharge are planned for later follow-up (2-3months). During this period, all patients completed at least one follow-up CT scan. We acknowledge that all patients who underwent CT scans during the follow-up process, especially those with substantial improvement in clinical manifestations, may raise concerns about radiation exposure.All subsequent chest CT results showed that the lung abnormalities had completely disappeared, and no patients reported respiratory symptoms during the follow-up period.

## 4 Discussion

Lipoid pneumonia was first described by Laughlin in 1925. It is a very rare lung disease caused by abnormal deposition of lipid substances in the lungs (7). Acute exogenous lipoid pneumonia (AELP) is mostly caused by inhaling large amounts of petroleum based products, usually occurring in children who are accidentally poisoned. The 12 cases collected in this study were all caused by accidental inhalation of oil substances, most of whom were under 3 years old. Some oil substances were contained in mineral water bottles without labeling, and the children mistakenly ingested them and developed the disease. This suggests that we should pay close attention to education on child protection awareness to prevent accidents from happening.

All patients in this study developed the disease due to aspiration of lipid substances such as sewing machine oil, electric mosquito repellent oil, or kerosene. These substances have the characteristics of high viscosity, difficulty in absorption, and the ability to destroy pulmonary surfactant. Inhalation can not only cause mechanical airway obstruction, but also induce local chemical inflammatory reactions, leading to increased alveolar capillary permeability, pulmonary edema, and inflammatory cell infiltration (8, 9). The clinical manifestations include non-specific respiratory symptoms such as cough, fever, and shortness of breath. Severe cases can rapidly progress to respiratory failure, which is consistent with the 5 cases in this study that required ventilator-associated ventilation.Respiratory distress and pulmonary hemorrhage are serious complications of childhood lipoid pneumonia, indicating widespread involvement and severe damage to the lungs.It is worth noting that children who have been exposed to oil for more than 12 h have lower oxygenation index, higher inflammatory indicators (WBC, CRP, LDH), longer hospitalization time,and are more prone to bacterial infections, suggesting that early medical treatment may be beneficial for suppressing the progression of the disease.

Exogenous lipoid pneumonia in children is prone to misdiagnosis in clinical practice. Without accurate medical history of the patient, the diagnosis of lipoid pneumonia is relatively difficult. The definitive diagnostic method of exogenous lipoid pneumonia is pathological biopsy (10). However, due to the difficulties in obtaining biopsy samples in pediatric clinical practice and the need for a certain amount of time for slide detection to obtain results, clinical diagnosis is first based on medical history and imaging findings. Therefore, imaging examinations play a rapid and important role in the diagnosis of this disease (11, 12).Nevertheless, we must acknowledge that the absence of pathological confirmation is a major limitation of this study. The diagnosis of AELP in our 12 cases was based solely on clinical, radiological, and bronchoscopic findings, which, while robust and widely accepted in clinical practice, do not replace the definitive diagnostic value of histopathology. According to relevant literature reports (13), pulmonary imaging changes in lipophilic pneumonia can occur as early as within 30 min of inhaling lipophilic substances and may progress within 24 h. The chest CT scans of the children included in this study within 24 h of admission showed exudation or consolidation, mainly involving both lower lungs, which is consistent with the anatomical distribution characteristics of inhalation lesions. High resolution CT can more sensitively display subtle changes such as ground glass opacities and bronchial inflation signs, providing important evidence for disease assessment. Fiber bronchoscopy can not only directly observe airway mucosal congestion, edema, and lipid secretions, but also obtain pathogenic evidence through alveolar lavage and perform therapeutic lavage. In this study, all positive cases of lavage fluid culture were in the delayed visit group group, further indicating that delayed treatment is prone to secondary infections, and bronchoscopy plays a dual role in diagnosis and treatment. This study also found that the oxygenation score of children with a hospital stay of more than 7 days was less than or equal to 7 days, and the difference was statistically significant, suggesting a potential association between low oxygenation score and prolonged hospitalization. However, due to the small sample size, multiple regression analysis could not be conducted. In the future, relevant samples will continue to be collected to lay the foundation for future research.

At present, there is no unified standard for effective treatment of AELP.In our case series, early fiberoptic bronchoscopy combined with corticosteroid therapy was associated with good clinical outcomes. However, due to the lack of a control group, we are unable to draw causal conclusions regarding the effectiveness of these interventions. But our observations are consistent with previous reports (14), indicating potential benefits. Lavage can directly remove lipid residues in the airway, alleviate local obstruction and inflammatory response (15); Corticosteroids can inhibit the release of inflammatory mediators, alleviate lung injury and fibrosis processes (16). All patients in this study received the above-mentioned comprehensive treatment, and 11 of them were cured.While these outcomes are encouraging, the lack of a control group precludes definitive conclusions about treatment efficacy. Especially for children who seek medical attention late and have high infection indicators, active anti infection and supportive treatment are equally indispensable. The overall prognosis of the children in this study was good, but there was still one death. This case had delayed medical treatment, extensive lung lesions, and concomitant bacterial infection, indicating that the timing of treatment, infection status, and extent of lesions are key factors affecting prognosis. According to relevant literature reports (17), the mortality rate of AELP has been reported to range from 13% to 50%,and the mortality rate in this group is relatively low, which may be related to early active intervention. Therefore, strengthening family care, avoiding children's exposure to flammable and easily aspirated substances, and immediately seeking medical attention in case of aspiration are fundamental measures to prevent and improve prognosis. This study has several limitations. Firstly, the 12 h cut-off time for group classification is based on the median interval of the study population, rather than validated clinical thresholds, which may introduce bias. Future research with larger sample sizes is needed to validate this discovery and establish more evidence-based threshold values. Secondly,this study is a single center retrospective analysis with limited sample size and lack of long-term follow-up data. We acknowledge that the statistically significant differences observed between the two groups must be interpreted with caution. These observed effect sizes are often unstable, so our results should be considered exploratory and hypothesis generative, and we should avoid generalizing these findings to a wider pediatric population.Further validation of the conclusions will require multi center and large sample studies in the future. Thirdly, the small sample size excluded multiple regression analysis to adjust for potential confounding factors.The type of oil, baseline disease severity, and use of mechanical ventilation may have affected the results, and all patients requiring mechanical ventilation belong to the delayed visit group, which may result in longer hospital stays and lower oxygenation scores in this group of patients. However, due to the limited sample size, we are unable to derive independent effects of delayed presentation from these confounding factors. In the future, more sample sizes are needed for corresponding analysis of confounding variables.Fourthly, this study did not include control group patients who did not receive bronchoscopy, corticosteroids, or antibiotics. Therefore, we cannot determine whether the observed favorable outcomes are attributed to the treatment itself, self recovery, or other factors. Any statement about therapeutic efficacy should be considered descriptive and hypothetical, rather than causal.Fifthly, considering the issue of radiation exposure, all patients in this study underwent chest CT follow-up upon discharge, especially those whose clinical manifestations had significantly improved before discharge. According to published reference levels for children, the estimated average effective radiation dose for a single chest CT scan in this age group (4-48 months) is approximately 2mSv (range 1.5-2.5mSv). Although this dosage is usually low and has minimal carcinogenic risk, it may not be strictly necessary for children with significantly improved symptoms and normal lung auscultation. The decision to conduct follow-up CT was mainly due to the retrospective nature of this study and the clinical doctor's desire to record complete absorption of imaging. However, following the ALARA principle, future prospective studies should adopt stricter follow-up imaging standards, such as only performing CT scans on patients with persistent or recurrent symptoms, abnormal lung signs, or suspected complications. For clinically improved children, alternative methods with lower or no radiation (such as chest x-rays or pulmonary ultrasound) may be considered for routine follow-up.In addition, AELP is rare in children and is prone to misdiagnosis or missed diagnosis in clinical practice. Improving awareness of this disease, inquiring about contact history in detail, and conducting imaging and bronchoscopy examinations in a timely manner are key to early diagnosis.

Acute exogenous lipoid pneumonia in children has a rapid onset and progression.In this limited sample, there is an association between delayed treatment and increased risk of lung injury and infection, but further research is needed to infer the causal relationship. A low oxygenation score may be a warning signal for prolonged hospitalization. Early identification and timely bronchoalveolar lavage combined with corticosteroid therapy may be beneficial, but prospective controlled studies are needed to confirm their efficacy. Medical history collection and imaging evaluation should be emphasized in clinical work, and health education for families should be strengthened to prevent the occurrence of such unexpected events.

## Data Availability

The raw data supporting the conclusions of this article will be made available by the authors, without undue reservation.
